# Bi-diketopyrrolopyrrole (Bi-DPP) as a novel electron accepting compound in low band gap π-conjugated donor–acceptor copolymers/oligomers

**DOI:** 10.1080/15685551.2016.1239173

**Published:** 2016-10-19

**Authors:** Johannes Ahner, Jürgen Nowotny, Ulrich S. Schubert, Martin D. Hager

**Affiliations:** ^a^ Laboratory of Organic and Macromolecular Chemistry (IOMC), Friedrich Schiller University Jena, Jena, Germany; ^b^ Jena Center for Soft Matter (JCSM), Friedrich Schiller University Jena, Jena, Germany

**Keywords:** Donor–acceptor polymers, diketopyrrolopyrrole, low band gap, organic solar cells, π-conjugated polymers

## Abstract

The synthesis and characterization of a novel 2,5-diketopyrrolo[3,4-*c*]pyrrole(DPP)-based accepting building block with the scheme DPP-neutral small linker-DPP (**Bi-DPP**) is presented, which was utilized as electron accepting moiety for low band gap π-conjugated donor–acceptor copolymers as well as for a donor–acceptor small molecule. The electron accepting moiety **Bi-DPP** was prepared *via* a novel synthetic pathway by building up two DPP moieties step by step simultaneously starting from a neutral phenyl core unit. Characterization of the synthesized oligomeric and polymeric materials via cyclic voltammetry afford LUMO energy levels from −3.49 to −3.59 eV as well as HOMO energy levels from −5.07 to −5.34 eV resulting in low energy band gaps from 1.52 to 1.81 eV. Spin coating of the prepared donor–acceptor oligomers/polymers resulted in well-defined films. Moreover, UV–vis measurements of the investigated donor–acceptor systems showed a broad absorption over the whole visible region. It is demonstrated that **Bi-DPP** as an electron accepting moiety in donor–acceptor systems offer potential properties for organic solar cell devices.

## Introduction

Since the development of the first solar cells in 1950s, there has been an intensive research on cost-effective and large scale industrial photovoltaics.[1] In particular, organic solar cells represent an interesting possibility to generate electric current from renewable natural sources due to their potential for cost-efficiency and large scale industrial production (e.g., roll-to-roll process).[2] During the last decade bulk hetero junction (BHJ) solar cells gained much attention in this field. In contrast to their inorganic counterpart, organic photovoltaics (OPV) offer several advantages like flexibility, lightweight and easy processing.[3] The ‘active material’ of such a BHJ solar cell consists generally of an interpenetrating network between a fullerene derivative acceptor compound and a π-conjugated low band gap organic donor polymer.[3] For instance, poly(3-hexylthiophene) (P3HT)-fullerene blends as active material in a BHJ solar cell have been investigated thoroughly for the past decade and yield power conversion efficiencies (PCEs) up to 5%, which can be explained by the good ability of phase separation of the blend and crystallization into desirable BHJ morphologies.[4–6] Another possibility to achieve a low band gap organic π-conjugated donor compound is the preparation of a π-conjugated donor–acceptor polymer. Typical electron-donating moieties for donor–acceptor polymers are cyclopenta[2,1-*b*:3,4-*b*′]dithiophene [7], benzo[1,2-*b*:4,5-*b*′]dithiophene [8] or fluorene derivatives [9]. In contrast, 2,5-diketopyrrolo[3,4-*c*]pyrrole (DPP) [10,11], benzodithiazole [12], [1,2,5]thiadiazolo[3,4-*g*]quinoxaline [13] or pyrazino[2,3-*g*]quinoxaline [14] represent well-known examples for electron-accepting compounds. In particular, the DPP moiety is one of the most widely studied acceptor units for high efficient low band gap polymers and, consequently, high PCEs of OPV devices.[15] Several DPP-based low band gap oligomers with the scheme DPP-Donor-DPP [11,16,17] as well as DPP-Donor-DPP-Donor-DPP [18] were investigated with PCEs up to 5.5%. Based on these considerations, here, a novel DPP-based accepting building block (**Bi-DPP**) with the scheme DPP-neutral linker-DPP is presented. The known donor core units between both DPP moieties were replaced by a short neutral linker, which leads to a larger π-conjugated acceptor building block, consisting of two DPP moieties. The prepared novel electron accepting moiety was introduced in a low band gap donor–acceptor small molecule as well as in donor–acceptor copolymers.

## Experimental section

### Materials and instrumentation

All chemicals and solvents were purchased from Sigma-Aldrich, Fluka, Acros Organics and Alfa Aesar and were used without further purifications. NMR spectra were recorded on a 300 MHz NMR spectrometer (Fourier 300) at 298 K and in deuterated solvents. Chemical shifts are reported in parts per million (ppm, *δ* scale) relative to the residual signal of the deuterated solvent. Elemental analyzes were performed on a *λ* EuroVector EuroEA3000 elemental analyzer. The reaction progress was monitored by thin layer chromatography using precoated aluminum sheets (silica gel 60 F254, Merck). Column chromatography was performed on silicagel 60 (pore size 60 Å, 70–230 mesh, 63–200 μm). ESI-TOF MS measurements were performed using a micrOTOF (Bruker Daltonics) mass spectrometer, which was equipped with a syringe pump for sample injection and a standard electrospray ion source. The mass spectrometer was operating in the positive ion mode and the data were processed with a micrOTOF control Version 3.0 and Data Analysis Version 4.0 SP2. CH_2_Cl_2_, acetonitrile and chloroform were used as solvents and the concentrations ranged from 1 to 10 μg/mL. The instrument was calibrated by a tunemix solution (50–3000 m/z) from Agilent. Bruker Ultraflex III MALDI TOF/TOF was used for MALDI-TOF MS measurements. SEC measurements of the copolymers were performed using a Shimadzu SCL-10A VP, DGU-14A as degasser, LC-10AD VP as the pump, a CTO-10A VP oven with 40 °C oven temperature, a SPD-10AD VP UV-detector, a RID-10A RI-detector, a PSS SDV guard/lin M column, a flow rate of 1 mL/min, THF + 1% diethylaminoethylamine as the eluent and a polystyrene calibration. Emission spectra were performed on a Jasco FP6500 spectrometer and the UV–vis spectra were measured on a PerkinElmer Lambda 45 UV–vis/NIR spectrometer. The measurements were carried out in solutions of chloroform (spectroscopic grade) at 25 °C in 1 cm cuvettes. The absorption/emission spectra of the prepared spin coated polymer films were measured by an UV–vis spectrometer Lamda 19/Perkin Elmer and a fluorescence spectrometer Hitachi F-4500. The electrochemical measurements were performed on a Metrohm Autolab PGSTAT30 potentiostat. A standard three-electrode setup was used, including a graphite-disk working electrode, a platinum-rod auxiliary electrode and a Ag/AgCl reference electrode. Ferrocene was used as internal standard. With the equation *E*_HOMO_/*E*_LUMO _= (−(*E*_onset _− *E*_onset,Fc/Fc+_) − 4.8) eV, the HOMO and LUMO energy levels were calculated. Atomic force microscopy (AFM) measurements were performed in tapping mode with a NTegra Aura (NT-MDT, Moscow, Russia) with commercially available non-contact cantilevers (NSC35, MicroMash). The scratches were made by using the lithographic mode of the AFM software by dragging the AFM tip at high forces along a line/cross over the surface.

### Synthesis of the monomers

#### Diethyl 3,3*′*-(1,4-phenylene)bis(3-hydroxypropanoate) (**1**)

To 155 mL anhydrous tetrahydrofuran, lithium diisopropylamide (82.89 mL, 1.8 M in hexane) was added in one portion at nitrogen atmosphere at −78 °C. Subsequently, anhydrous ethyl acetate (14.70 mL, 149.21 mmol) was added dropwise within 1.5 h. After stirring for 1 h terephthalaldehyde (10.00 g, 74.60 mmol), dissolved in 100 mL anhydrous tetrahydrofuran, was injected in one portion. The yellowish suspension was allowed to stir for additional 3 h at −78 °C. Afterwards the reaction mixture was quenched by adding 300 mL saturated ammonium chloride solution. The organic layer was separated and the aqueous phase was extracted two times with 100 mL dichloromethane. The combined organic phases were washed two times with 100 mL distilled water and once with brine. After drying over magnesium sulfate, the solvent was removed under reduced pressure. Subsequently, the crude product was purified by column chromatography with dichloromethane:ethyl acetate (10:1) to obtain a yellow oil with a yield of 13.74 g (59%). ^1^H NMR (CD_2_Cl_2_, *δ*) 1.24 (t, *J* = 7.1 Hz, 6H, CH_3_), 2.64–2.76 (m, 4H, O=CCH_2_), 3.29 (d, *J* = 3.5 Hz, 2H, OH), 4.12–4.19 (m, 4H, OCH_2_), 5.07–5.22 (m, 2H, OH–CH), 7.36 (s, 4H, Ph) ppm; ^13^C NMR (CDCl_3_, *δ*): 14.2, 43.3, 60.9, 70.0, 125.9, 142.1, 172.4 ppm; HR-ESI-TOF MS: calcd.: M = 333.1309 g/mol; found: m/z = 329.1309 ([M] ^+^), error: 0.1 ppm.

#### Diethyl 3,3*′*-(1,4-phenylene)bis(3-oxopropanoate) (**2**)

A suspension of compound **1** (13.38 g, 43.14 mmol), pyridinium chlorochromate (23.25 g, 107.85 mmol) and celite (38.63 g) in 400 mL dichloromethane was stirred overnight at room temperature for 12 h. Subsequently, the solvent was removed under reduced pressure and the obtained dark green solid was extracted five times with 100 mL ethyl acetate. After the solvent of the combined organic phases was removed under reduced pressure, the crude product was purified by column chromatography with heptane:ethyl acetate (3:1). Finally, a light yellow powder was obtained with a yield of 4.07 g (33%). *T*_m_ = 70 °C; ^1^H NMR (CDCl_3_, *δ*) 1.24–1.36 (two partially overlapped triplets, ^3^ *J* = 7.07 Hz, 6H, CH_3_), 4.00–4.02 (two partially overlapped singlets, 1.45H, O=CCH_2_), 4.19-4.31 (two partially overlapped quartets, 4H, OCH_2_), 5.71–5.74 (two singlets, 1.28H, C=CH), 7.82 (s, 1.47H, Ph), 7.87 (d, *J* = 8.8 Hz, 1.05H, Ph), 7.99 (d, *J* = 8.8 Hz, 1.06H, Ph), 8.04 (s, 0.38H, Ph), 12.55–12.56 (two partially overlapped singlets, 1.25H, OH) ppm; ^13^C NMR (CDCl_3_, *δ*): 14.1, 14.3, 14.3, 46.1, 46.2, 60.1, 60.1, 61.6, 61.7, 88.4, 89.4, 126.2, 126.4, 128.7, 128.8, 135.9, 137.7, 138.2, 139.5, 167.0, 167.2, 169.5, 170.1, 172.8, 173.0, 191.9, 191.9 ppm; HR-ESI-TOF MS: calcd.: M = 329.0996 g/mol; found: m/z = 329.0989 ([M] ^+^)^+^), error: 1.9 ppm; Elemental analysis: calcd. for C_16_H_18_O_6_: C 62.74, H 5.92; found: C 63.12, H 6.13.

#### Tetraethyl 2,2*′*-terephthaloyldisuccinate (**3**)

Compound **2** (10.25 g, 33.47 mmol), potassium carbonate (9.71 g, 70.28 mmol), sodium iodide (1.25 g, 8.37 mmol) and ethyl chloroacetate (8.41 g, 68.60 mmol) were dissolved in 60 mL acetone and 40 mL 12-dimethoxyethane. The reaction mixture was stirred 24 h at 75 °C. Subsequently, the solution was filtered and the solvent was removed under reduced pressure. Afterwards, 100 mL dichloromethane were added and the solution was washed twice with brine. The mixture was dried over sodium sulfate and the solvent was removed under reduced pressure again. After drying under vacuum an orange oil was obtained with a yield of 15.41 g (96%). ^1^H NMR (CD_2_Cl_2_, *δ*) 1.10–1.23 (m, 12H, CH_3_), 2.95–3.34 (m, 4H, O=CCH_2_), 4.01–4.21 (m, 8H, OCH_2_), 4.82–4.88 (m, 1.4H, O=CCH), 7.73–8.13 (m, 4H, Ph), 9.99–10.11 (three singlets, 0.37H, OH) ppm. ^13^C NMR (CD_2_Cl_2_, *δ*): 13.6, 13.8, 13.9, 33.2, 38.4, 49.7, 49.8, 58.1, 58.2, 60.9, 61.1, 61.9, 62.5, 128.0, 128.3, 128.6, 129.0, 130.0, 138.3, 139.6, 141.1, 168.2, 169.8, 170.3, 180.0, 193.9, 194.0, 196.2 ppm; ESI-TOF MS: calcd.: M = 478.18 g/mol; found: m/z = 501.17 ([M + Na] ^+^); Elemental analysis: calcd. for C_24_H_30_O_10_: C 60.24, H 6.32; found: C 60.17, H 6.24.

#### Diethyl 2,2*′*-(1,4-phenylene)bis(5-oxo-4,5-dihydro-1H-pyrrole-3-carboxylate) (**4**)

Compound **3** (9.89 g, 20.67 mmol) and ammonium acetate (30.28 g, 392.79 mmol) were dissolved in 120 mL anhydrous acetic acid under nitrogen atmosphere and the resulting reaction mixture was stirred 19 h at 120 °C. Subsequently, the reaction mixture was cooled to room temperature and afterwards poured into 300 mL ice water. The precipitate was filtered and washed several times with 20 mL ethyl acetate, *N*,*N*-dimethylformamide and dichloromethane. After the product was dried under vacuum, a light gray solid was obtained with a yield of 4.31 g (54%). *T*_m_ > 320 °C; ^1^H NMR (DMSO-d6, *δ*) 1.11 (t, *J* = 7.1 Hz, 6H, CH_3_), 3.41 (s, 4H, O=CCH_2_), 4.02 (q, *J* = 7.1 Hz, 4H, OCH_2_), 7.64 (s, 4H, Ph), 10.76 (s, 2H, NH) ppm; ^13^C NMR (DMSO-d6, *δ*): 14.5, 38.8, 59.7, 103.9, 128.9, 131.5, 152.3, 163.1, 178.0 ppm; MALDI-TOF MS: calcd.: M = 384.13 g/mol; found: m/z = 407.22 ([M + Na] ^+^); Elemental analysis: calcd. for C_20_H_20_N_2_O_6_: C 62.49, H 5.24, N 7.29; found: C 61.82, H 5.27, N 7.17.

#### 6,6*′*-(1,4-Phenylene)bis(3-(thiophen-2-yl)pyrrolo[3,4-c]pyrrole-1,4(2H,5H)-dione) (**5**)

Sodium (4.08 g, 177.91 mmol) was slowly added to 140 mL anhydrous amyl alcohol under a nitrogen atmosphere while the reaction mixture was slowly heated to 120 °C. After all sodium was dissolved, the solution was cooled to room temperature. Subsequently, compound **4** (9.00 g, 23.41 mmol) and thiophenecarbonitrile (6.54 mL, 70.23 mmol) were added to the reaction mixture in one portion. The solution was stirred for additionally 18 h at 80 °C. After cooling to room temperature, a mixture of 250 mL methanol and 30 mL acetic acid was added and the resulted suspension was stirred for additional 30 min. The precipitate was filtered and washed several times with 50 mL distilled water, 50 mL methanol and 50 mL dichloromethane. After drying under reduced pressure, a dark purple solid was obtained with a yield of 11.66 g (98%). The crude product was used without further purification.

#### 6,6*′*-(1,4-Phenylene)bis(2,5-bis(2-ethylhexyl)-3-(thiophen-2-yl)pyrrolo [3,4-c]pyrrole-1,4(2H,5H)-dione) (**6**)

The crude product of compound **5** (11.60 g, 22.74 mmol) and potassium carbonate (25.14 g, 181.92 mmol) were dissolved in 200 mL dry dimethyl sulfoxide and stirred at 100 °C for 3 h under nitrogen atmosphere. Subsequently, 2-ethylhexyl iodide (49.02 mL, 272.91 mmol) was added in one portion and the reaction mixture was stirred for additional 19 h at 100 °C. After cooling to room temperature, the reaction mixture was poured into 400 mL ice water and stirred for one hour. The precipitation was filtered and extracted several times with ethyl acetate. The insoluble part was washed several times with 50 mL distilled water, 50 mL methanol and 50 mL heptane. After drying under vacuum a dark purple solid was obtained, which was used as raw material for repeating the whole procedure four times. Subsequently, the solvent of the whole combined extracted organic phases (ethyl acetate) from the repeated procedures was removed under reduced pressure. Afterwards, the crude product was purified via column chromatography with heptane:ethyl acetate (9:1) to obtain a dark purple solid with an overall yield of 2.49 g (11%). *T*_m_ = 227 °C; ^1^H NMR (CD_2_Cl_2_, *δ*) 0.65–0.98 (m, 24H, CH_3_), 1.00–1.57 (m, 32H, CH_2_; m, 2H, N–CH_2_–CH), 1.82 (m, 2H, N–CH_2_–CH), 3.78 (m, 4H, N–CH_2_), 4.00 (m, 4H, N–CH_2_), 7.10 (t, *J* = 3.9 Hz, 2H, Ar-thio), 7.63 (d, *J* = 4.7 Hz, 2H, Ar-thio), 7.80 (s, 4H, Ph), 8.71 (d, *J* = 2.4 Hz, 2H, Ar-thio) ppm. ^13^C NMR (CD_2_Cl_2_, *δ*): 10.1, 10.2, 13.7, 13.8, 22.3, 22.8, 23.1, 23.5, 28.3, 28.3, 28.5, 30.3, 38.4, 38.6, 39.0, 44.8, 45.5, 107.8, 110.0, 128.3, 128.8, 129.8, 130.2, 131.2, 135.7, 142.3, 145.2, 161.5, 162.1 ppm; HR-ESI-TOF MS: calcd.: M = 959.5537 g/mol; found: m/z = 959.5484 ([M] ^+^), error: 5.6 ppm.

#### 6,6*′*-(1,4-Phenylene)bis(3-(5-bromothiophen-2-yl)-2,5-bis(2-ethylhexyl)pyrrolo[3,4-c]pyrrole-1,4(2H,5H)-dione) (**Bi-DPP**)

Compound **6** (2.21 g, 2.31 mmol) was dissolved in 45 mL anhydrous chloroform under nitrogen atmosphere. Afterwards bromine (0.23 mL, 5.76 mmol), dissolved in 11 mL anhydrous chloroform, was added dropwise over one hour at room temperature. Subsequently, the reaction mixture was allowed to stir for additional four hours. Afterwards, the solvent was removed under reduced pressure and the resulted crude product was purified by column chromatography with hexane:ethyl acetate (8:1). Finally, a dark purple solid was obtained with a yield of 1.51 g (59%). *T*_m_ = 259 °C; ^1^H NMR (CD_2_Cl_2_, *δ*) 0.76 (t, *J* = 7.1 Hz, 24H, CH_3_), 0.81–1.46 (m, 32H, CH_2_; m, 2H, N–CH_2_–CH), 1.74 (m, 2H, N–CH_2_–CH), 3.66 (m, 4H, N–CH_2_), 3.90 (m, 4H, N–CH_2_), 6.98 (d, *J* = 4.1 Hz, 2H, Ar-thio), 7.61 (s, 4H, Ph), 8.29 (d, *J* = 4.1 Hz, 2H, Ar-thio) ppm. ^13^C NMR (CD_2_Cl_2_, *δ*): 10.6, 10.7, 14.2, 14.4, 23.4, 23.6, 23.9, 28.8, 29.0, 30.8, 38.4, 38.7, 39.4, 45.0, 46.0, 108.5, 110.0, 120.0, 129.4, 130.5, 131.6, 131.9, 136.0, 141.4, 146.5, 161.7, 162.4 ppm; MALDI-TOF MS: calcd.: M = 1114.37 g/mol; found: m/z = 1114.02 ([M] ^+^); Elemental analysis: calcd. for C_58_H_76_Br_2_N_4_O_4_S_2_: C 62.35, H 6.86, N 5.01, S 5.74, Br 14.30; found: C 62.61, H 7.05, N 4.91, S 5.58, Br 14.38.

#### 3,6-Bis(5-(benzofuran-2-yl)thiophen-2-yl)-2,5-bis(2-ethylhexyl)pyrrolo[3,4-c]pyrrole-1,4(2H,5H)-dione (**SM-Bi-DPP**)

**Bi-DPP** (100.00 mg, 0.1465 mmol), 2-benzofuranyl boronic acid (53.38 mg, 0.3296 mmol), tris(dibenzylideneacetone)dipalladium(0) (6.70 mg, 0.0073 mmol), tri-*t*-butylphosphonium tetrafluoroborate (7.22 mg, 0.0249 mmol) and potassium phosphate (621.98 mg, 2.9300 mmol) were dissolved in a mixture of 7 mL anhydrous tetrahydrofuran and 1.5 mL distilled water under nitrogen atmosphere. Subsequently, the reaction mixture was stirred overnight at room temperature. Afterwards, the dark blue solution was extracted several times with chloroform. The combined organic fractions were washed with 50 mL distilled water and subsequently the solvent was removed under reduced pressure. The crude product was dissolved in 3 mL chloroform and precipitated in 60 mL methanol. Subsequently, the precipitate was filtered. After the crude product was purified by flash chromatography with chloroform, a dark blue solid was obtained with a yield of 159 mg (91%). *T*_m_ = 258 °C; ^1^H NMR (CDCl_3_, *δ*) 0.55–0.98 (m, 24H, CH_3_), 1.00–1.50 (m, 32H, CH_2_; m, 2H, N–CH_2_–CH), 1.84 (m, 2H, N–CH_2_–CH), 3.76 (m, 4H, NCH_2_), 4.08 (m, 4H, NCH_2_), 6.86 (s, 2H, Ar-fur), 7.16 (d, *J* = 3.4 Hz, 2H, Ar-thio), 7.19–7.47 (m, 8H, Ar-Ph), 7.68 (s, 4H, Ar-Ph), 8.56 (d, *J* = 3.7 Hz, 2H, Ar-thio) ppm; ^13^C NMR (CDCl_3_, *δ*): 10.5, 10.8, 14.3, 14.4, 23.6, 23.7, 28.9, 29.3, 30.7, 38.7, 39.5, 45.1, 103.9, 108.9, 110.2, 111.6, 221.7, 124.0, 125.7, 125.8, 129.4, 129.5, 130.4, 130.5, 137.0, 138.8, 141.9, 146.1, 150.8, 155.4, 161.7, 162.6 ppm; MALDI-TOF MS: calcd.: M = 1190.60 g/mol; found: m/z = 1190.31 ([M] ^+^); Elemental analysis: calcd. for C_74_H_86_N_4_O_6_S_2_: C 74.59, H 7.27, N 4.70, S 5.38; found: C 74.62, H 7.37, N 4.71, S 5.34.

### Synthesis of the polymers

#### Poly((**7**-**F1**)-stat-(**F2**-**F1**)) (**P1**)

**Bi-DPP** (100.00 mg, 0.090 mmol), 22′-(9,9′-dioctylfluorene-2,7-diyl)-bis(1,3,2-dioxaborolane) (**F1**) (105.24 mg, 0.180 mmol), 2,7-dibromo-9,9-dioctyl-9H-fluorene (**F2**) (49.21 mg, 0.090 mmol), potassium carbonate (119.05 mg, 0.861 mmol), 3 mol% Pd(PPh_3_)_4_ and two droplets of Aliquat 336© were dissolved in 9 mL toluene and 1 mL distilled water under nitrogen atmosphere. Additionally, the solution was purged 30 min with nitrogen. Subsequently, the mixture was stirred at 120 °C for 48 h. After the solution was cooled to room temperature a mixture of 10 mL distilled water and 10 mL chloroform was added. The organic phase was washed twice with 10 mL distilled water and, subsequently, the solvent was removed under reduced pressure. The crude product was dissolved in 3 mL chloroform and precipitated in methanol (1:20). The precipitation was filtered and washed several times with cold methanol. Copolymer **P1** was obtained as a dark blue solid with a yield of 167 mg (84%). ^1^H NMR (CDCl_3_, *δ*): 0.50–1.70 (m, CH_3_; m, CH_2_), 1.82 (m, NCH_2_CH), 2.10 (m, CCH_2_), 3.00–4.40 (m, NCH_2_), 7.30–8.30 (m, Ar-Thio; m, Ar-Ph), 9.10 (m, Ar-Thio) ppm; SEC (THF, PS calibration): *M*_n_ = 9000 g/mol, *M*_w_ = 19,200 g/mol, *Ð* = 2.1.

#### Poly((**7**-**S1**)-stat-(**S2**-**S1**)) (**P2**)

**Bi-DPP** (100.00 mg, 0.090 mmol), 5,5-dioctyl-3,7-bis(4,4,5,5-tetramethyl-1,3,2-dioxaborolan-2-yl)-5H-dibenzo[b,d]silole (**S1**) (118.20 mg, 0.180 mmol), 3,7-dibromo-5,5-dioctyl-5H-dibenzo[b,d]silole (**S2**) (50.65 mg, 0.090 mmol), potassium carbonate (119.05 mg, 0.861 mmol), 3 mol% Pd(PPh_3_)_4_ and two droplets of Aliquat 336© were dissolved in 9 mL toluene and 1 mL distilled water under nitrogen atmosphere. Further steps were prepared according to the procedure of copolymer **P1** to obtain copolymer **P2** as a dark blue solid with a yield of 197 mg (99%). ^1^H NMR (CDCl_3_, *δ*): 0.50–1.50 (m, CH_3_; m, CH_2_), 1.59 (m, NCH_2_CH), 1.85 (m, NCH_2_CH), 3.11–4.20 (m, NCH_2_), 7.90–8.20 (m, Ar-Thio; m, Ar-Ph), 9.05 (m, Ar-Thio) ppm; SEC (THF, PS calibration): *M*_n_ = 10,800 g/mol, *M*_w_ = 33,700 g/mol, *Ð* = 3.1.

#### Poly((**7**-**S1**)-stat-(**B1**-**S1**)) (**P3**)

**Bi-DPP** (100.00 mg, 0.090 mmol), 5,5-dioctyl-3,7-bis(4,4,5,5-tetramethyl-1,3,2-dioxaborolan-2-yl)-5H-dibenzo[b,d]silole (**S1**) (118.20 mg, 0.180 mmol), 2,6-dibromo-4,8-bis(octyloxy)benzo[1,2-b:4,5-b′]dithiophene (**B1**) (49.21 mg, 0.090 mmol), potassium carbonate (119.05 mg, 0.861 mmol), 3 mol% Pd(PPh_3_)_4_ and two droplets of Aliquat 336© were dissolved in 9 mL toluene and 1 mL distilled water under nitrogen atmosphere. Further steps were prepared according to the procedure of copolymer **P1** to obtain copolymer **P3** as a green solid with a yield of 188 mg (95%). ^1^H NMR (CDCl_3_, *δ*): 0.50–1.50 (m, CH_3_; m, CH_2_), 1.59 (m, NCH_2_CH), 1.85 (m, NCH_2_CH), 3.11–4.20 (m, NCH_2_), 7.90–8.20 (m, Ar-Thio; m, Ar-Ph), 9.05 (m, Ar-Thio) ppm; SEC (THF, PS calibration): *M*_n_ = 15,500 g/mol, *M*_w_ = 64,300 g/mol, *Ð* = 4.2.

#### Poly((**7**-**B2**)-stat-(**S2**-**B2**)) (**P4**)

**Bi-DPP** (100.00 mg, 0.090 mmol), (49.21 mg, 0.090 mmol), 3,7-dibromo-5,5-dioctyl-5H-dibenzo[b,d]silole (**S2**) (50.65 mg, 0.090 mmol), (4,8-bis(octyloxy)benzo[1,2-b:4,5-b′]dithiophene-2,6-diyl)bis(trimethylstannane) (**B2**) (138.60 mg, 0.180 mmol) and 3 mol% Pd(PPh_3_)_4_ were dissolved in 9 mL toluene under nitrogen atmosphere. Additionally, the solution was purged 30 min with nitrogen. Subsequently, the mixture was stirred at 100 °C for 48 h. After the solution was cooled to room temperature the solution was dropwise precipitated in 100 mL methanol. The precipitation was filtered and washed several times with 50 mL distilled water, 50 mL methanol and 50 mL ethyl acetate. Finally, copolymer **P4** was obtained as a dark green solid with a yield of 162 mg (80%). ^1^H NMR (CDCl_3_, *δ*): 0.50–2.20 (m, CH_3_; m, CH_2_), 4.01 (m, NCH_2_), 4.27 (m, OCH_2_), 6.50–8.00 (m, Ar-Thio; m, Ar-Ph), 8.63 (m, Ar-Thio) ppm; SEC (THF, PS calibration): *M*_n_ = 31,400 g/mol, *M*_w_ = 138,500 g/mol, *Ð* = 4.4.

## Results and discussion

### Synthesis of the electron accepting building block Bi-DPP

**Scheme 1.**
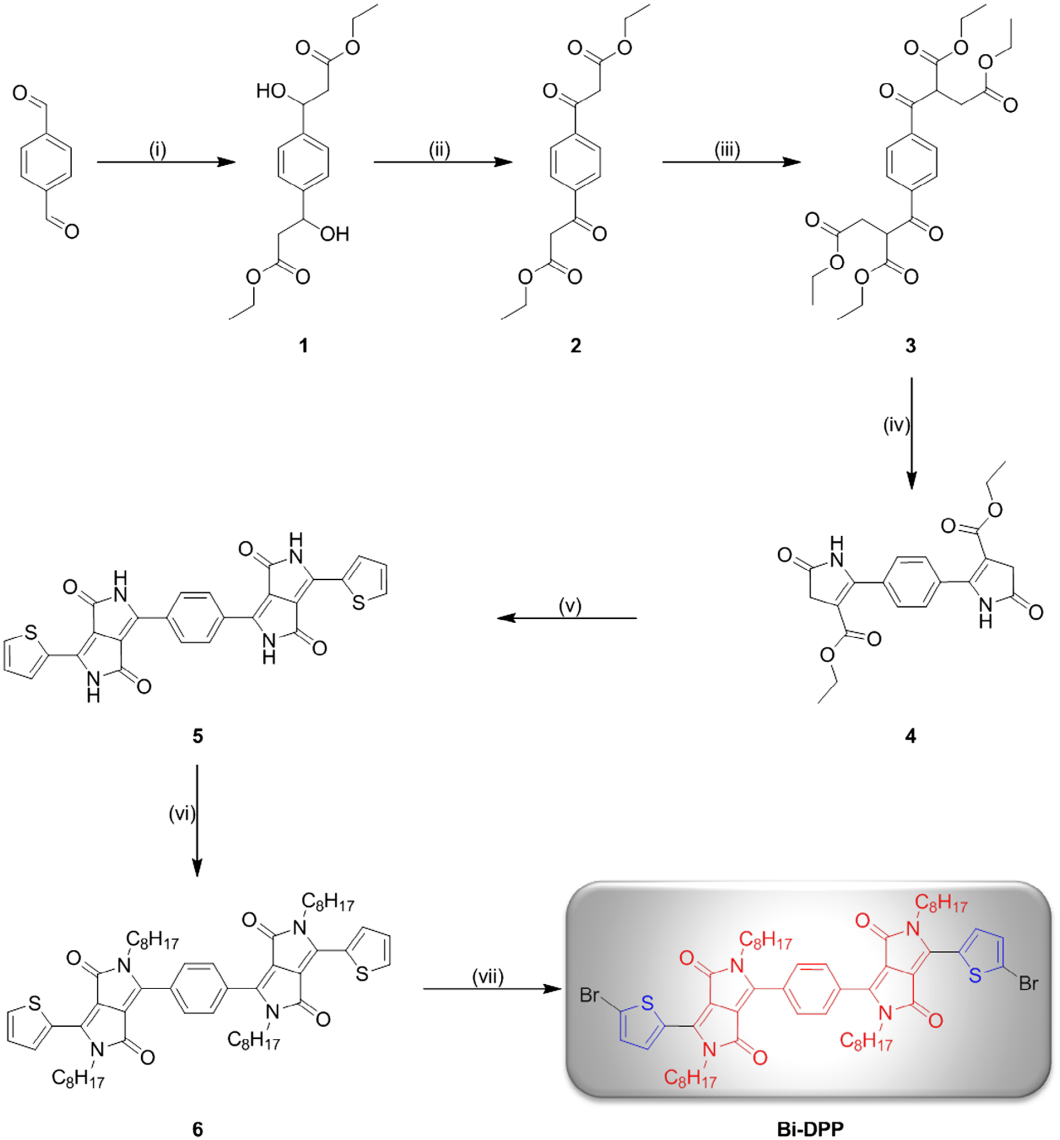
Schematic representation of the synthesis of **Bi-DPP**. Reagents and conditions: (i) Ethyl acetate/lithium diisopropylamide/tetrahydrofuran/−78 °C; (ii) pyridinium chlorochromate/celite/dichloromethane/25 °C; (iii) ethyl 2-chloroacetate/potassium carbonate/sodium iodide/acetone/55 °C; (iv) ammonium acetate/acetic acid/120 °C; (v) thiophene-2-carbonitrile/Na-*t*-OC_5_H_12_/80 °C; (vi) 2-ethylhexyl iodide/potassium carbonate/dimethyl sulfoxide/100 °C; and (vii) bromine/chloroform/25 °C.

In order to prepare a DPP-based electron accepting building block with the general motif DPP-Core-DPP, several different approaches are possible. However, to the best of our knowledge, DPP-Core-DPP- or DPP-Core-DPP-Core-DPP-based structures were only synthesized by coupling the DPP moieties using several cross coupling techniques. For instance Marks and co-workers as well as Huang et al*.* coupled two DPP moieties with one typical donor core unit via Stille cross coupling reaction in order to obtain small molecules for organic solar cell devices with the scheme Donor-DPP-Donor-DPP-Donor.[11,16] Moreover, small molecules, which exhibit the structure Donor-DPP-Donor-DPP-Donor-DPP-Donor, were synthesized via Suzuki or Stille cross coupling reaction by the groups of Nguyen and Li [17,18]. In contrast, the synthesis of a Donor-DPP-Core-DPP-Donor type with a small neutral core unit (e.g., phenyl) is rather difficult using cross coupling reactions. Based on these considerations, the synthesis of a novel **Bi-DPP** was implemented by building up two DPP units step by step starting from a neutral phenyl core (Scheme 1). Compound **1** was synthesized via an aldol addition with ethyl acetate and lithium diisopropylamide in tetrahydrofuran at low temperatures of −78 °C with a yield of 59%. Subsequently, the treatment of the alcohol groups of compound **1** with pyridinium chlorochromate and celite in dichloromethane at room temperature afforded compound **2** and the possible tautomers in a yield of 33%. Substitution reaction of compound **2** with ethyl 2-chloroacetate and potassium carbonate in acetone afforded intermediate **3** in a yield of 96%. The first ring closure of the DPP unit on both sides of the phenyl ring was implemented with ammonium acetate under acetic acid conditions at 120 °C leading to compound **4** with a yield of 54%. After the second ring closure, the desired Donor-DPP-Phenyl-DPP-Donor **5** structure was prepared. Due to its slight solubility in common organic solvents, the crude product was utilized for the further step. In order to encounter this insoluble trend, compound **5** was alkylated at each amide position with an ethylhexyl alkyl chain by a simple substitution reaction of compound **5** with 2-ethylhexyl iodide and potassium carbonate in dimethyl sulfoxide at 100 °C. This alkylation step was repeated four times to afford compound **6** with a low yield of 11%, which is due to the fact that mono-, di- and tri-alkylated as well as *O*-alkylated side products are possible.[19] Noteworthy, the alkylation of classical DPPs also results in low yields. The final **Bi-DPP** was achieved in a yield of 59% with a single bromination at each thiophene moiety in chloroformic solution at room temperature. For the first time, two DPP moieties were build up step by step from a simple phenyl core unit. For further investigations, the core unit can be exchanged by several simple conjugated systems (e.g., furan, thiophene, and pyrrole) and the same synthetic procedure can be implemented to achieve an electron accepting building block library.

### Synthesis and characterization of the prepared Bi-DPP-based conjugated donor–acceptor copolymers and small molecules

**Figure 1.**
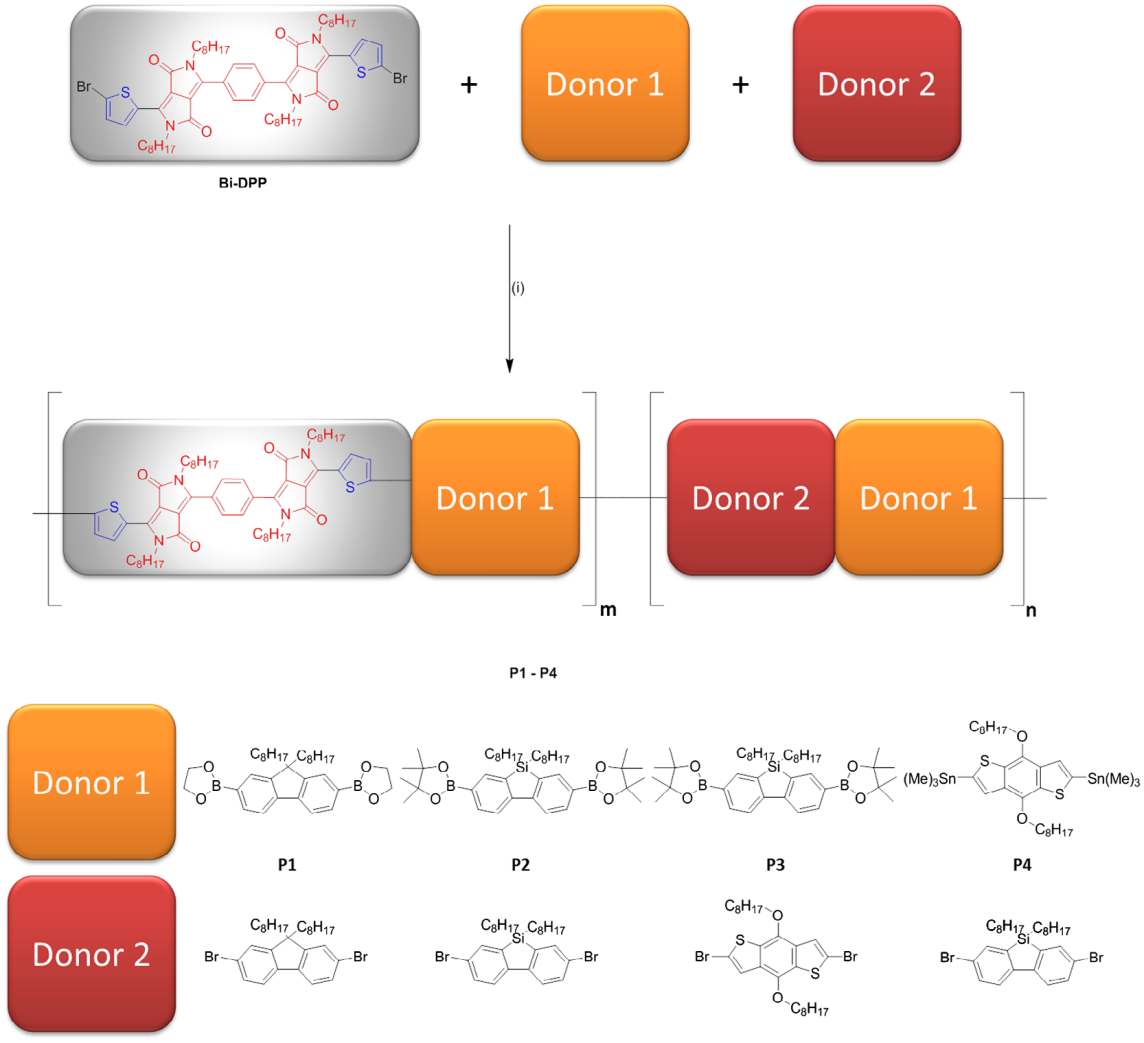
Synthetic route of **P1**–**P4**. Reagents and conditions: (i) In case of the Suzuki cross coupling reaction: Pd(PPh_3_)_4_/potassium carbonate/Aliquat 336©/water/toluene/120 °C; in case of the Stille cross coupling reaction: Pd(PPh_3_)_4_/toluene/100 °C.

The prepared novel electron accepting building block **Bi-DPP** was polymerized via Suzuki or Stille cross coupling reactions with several different typical donor moieties in order to achieve potential donor–acceptor copolymers as an active material for organic solar cell devices. The general synthetic procedure is outlined in Figure 1. Hereby, Pd(PPh_3_)_4_ serves as the catalyst and Aliquat 336© as the phase transfer catalyst in a solvent mixture of aqueous potassium carbonate and toluene. First, **Bi-DPP** was polymerized with 2,2′-(9,9′-dioctylfluorene-2,7-diyl)-bis(1,3,2-dioxaborolane) (**F1**), 5,5-dioctyl-3,7-bis(4,4,5,5-tetramethyl-1,3,2-dioxaborolan-2-yl)-5H-dibenzo[b,d]silole (**S1**) or (4,8-bis(octyloxy)benzo[1,2-b:4,5-b′]dithiophene-2,6diyl)bis(trimethylstannane) (**B2**), which leads to completely insoluble donor–acceptor copolymers with a low molar mass. Consequently, those prepared copolymers were unsuitable for commercial roll-to-roll processed organic solar cells. Therefore, the content of the more flexible donor moiety, connected with a better solubility, was increased. According to this, copolymer **P1** was synthesized via Suzuki cross coupling reaction with **F1** and 2,7-dibromo-9,9-dioctyl-9H-fluorene (**F2**) (**P1**:**F1**:**F2**, 0.5:1:0.5), whereby a soluble statistical donor–acceptor copolymer with a suitable molar mass (*M*_n_ = 9000 g/mol) was achieved.

**Figure 2.**
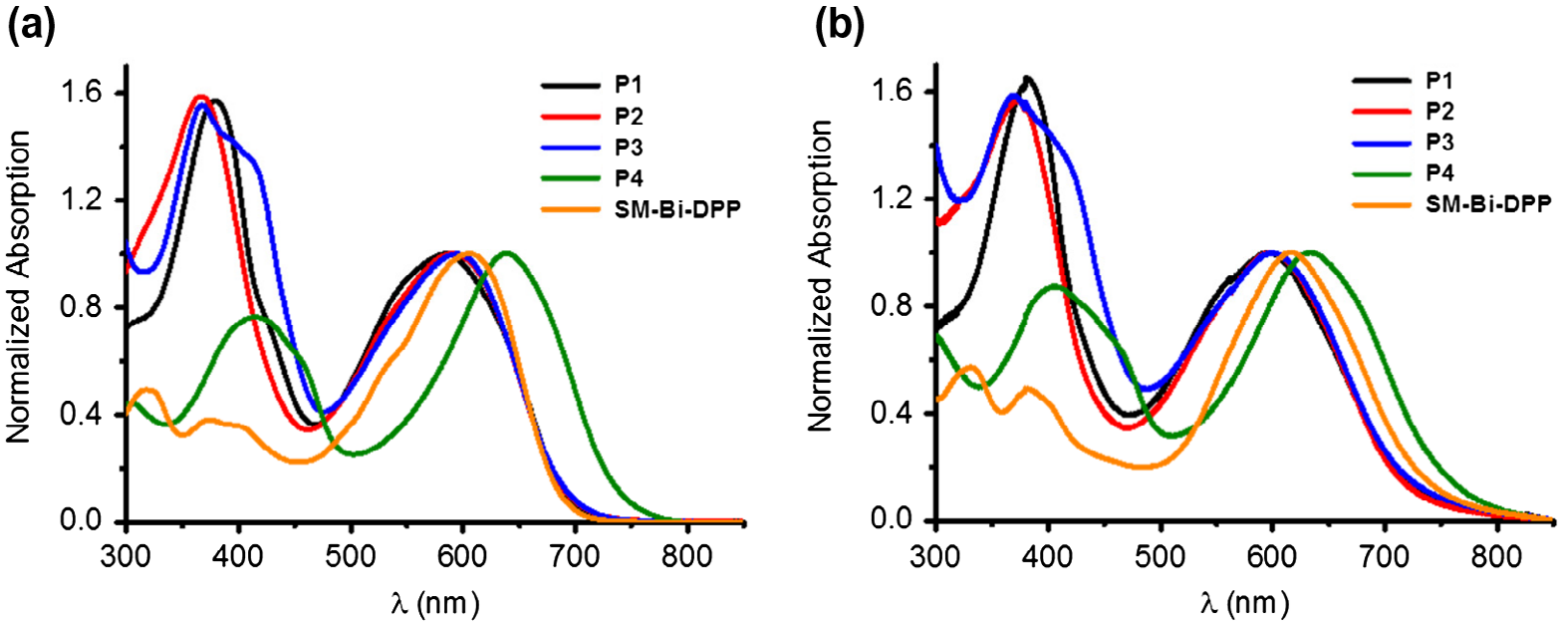
Normalized absorption spectra of the prepared copolymers **P1**–**P4** and small molecule **SM-Bi-DPP** (a) in chloroform and (b) as thin film.

**Figure 3.**
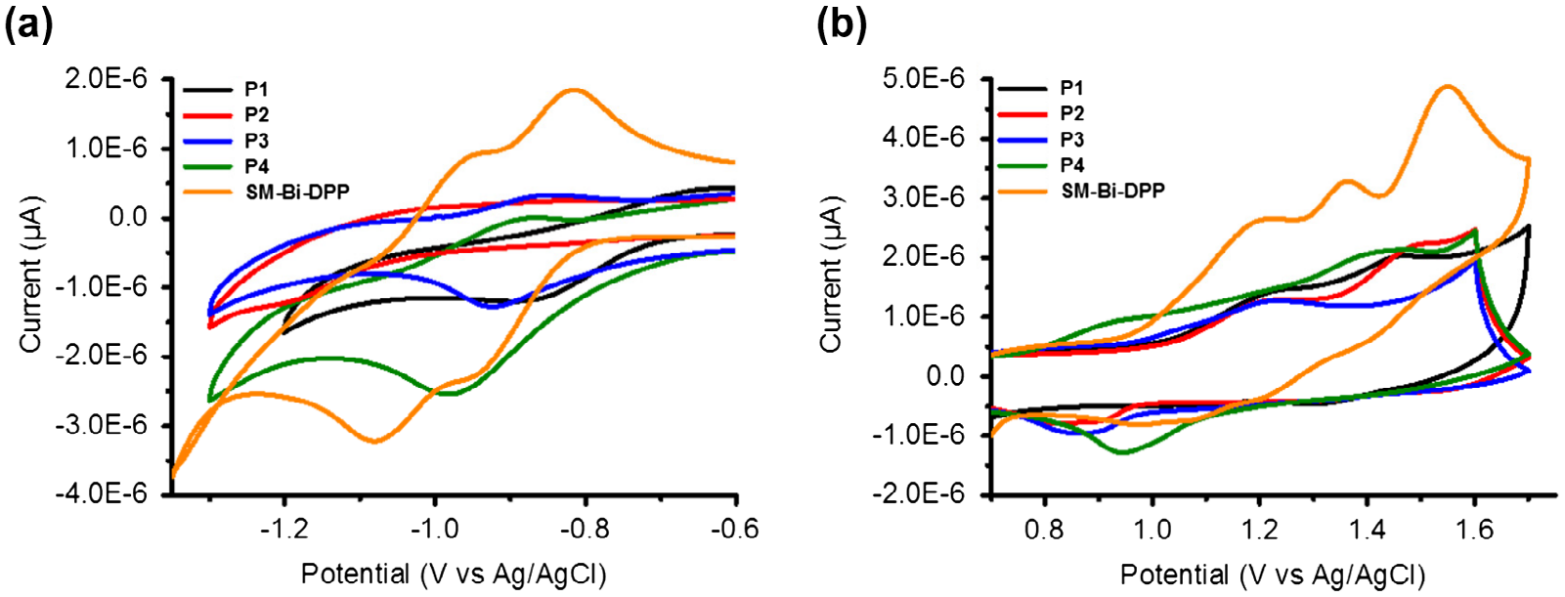
Cyclic voltammograms of the prepared copolymers and the small molecule: (a) Reduction and (b) oxidation.

**Figure 4.**
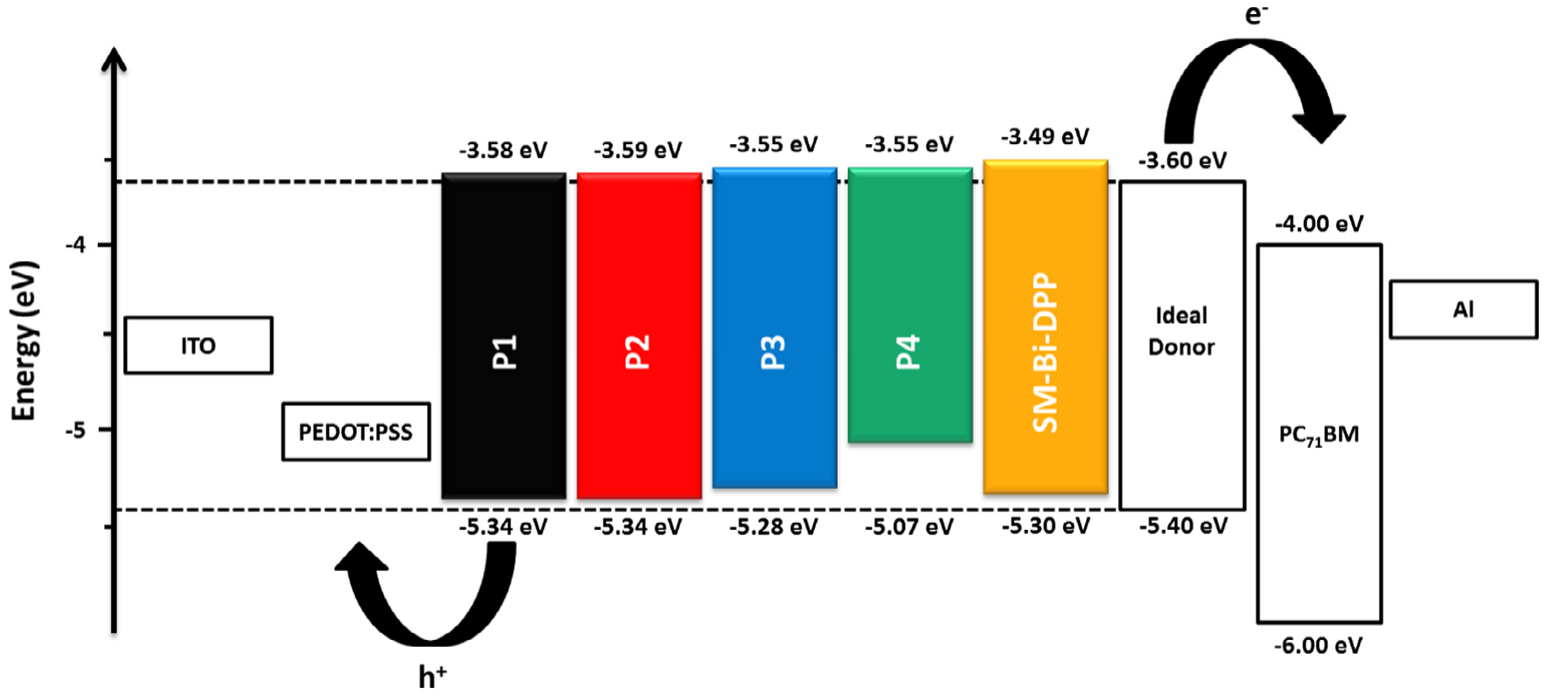
Illustration of the energy level diagrams of the prepared copolymers as well as of the small molecule.

Copolymer **P2** was prepared with the same procedure as copolymer **P1**, whereas the bridging carbon atom of the fluorene unit was exchanged by a silicon. The resulting silacyclopentadiene moiety has been investigated as a system where the interaction of the π*-orbital of the butadiene fragment with the σ*-orbital of the silicon–carbon bond leads to a lower LUMO energy level.[20] Furthermore, this interaction results in good electron mobility and affinity. Silafluorene-based donor–acceptor copolymers exhibit low lying HOMO energy levels and high open voltage circuits, which resulted in PCEs up to 6.41%.[21] Furthermore, the typical donor unit 4,8-bis(octyloxy)benzo[1,2-b:4,5-b′]dithiophene-2,6-diyl(**B**) was utilized for copolymerization with the prepared **Bi-DPP**. Due to the increased donor strength of **B** in contrast to fluorene derivatives, the HOMO energy level of the donor–acceptor copolymer should increase, which finally leads to lower band gap energies. First, **B** was deliberately introduced into copolymer **P3**, whereby **B** is always separated from the acceptor building block **Bi-DPP** by suitable bisbromo-functionalization of **B**. In contrast, bistrimethylstannyl-functionalization of **B** leads to copolymer **P4**, whereby **B** is located always directly next to **Bi-DPP** resulting in a stronger interaction between **B** and **Bi-DPP**. The achieved donor–acceptor copolymers were characterized by ^1^H NMR spectroscopy and size exclusion chromatography. The resulting molar mass and dispersiy (*Ð*) values are summarized in Table 1, whereby the dispersity ranges from 2.1 to 4.4, which is typical for polycondensation reactions.

**Table 1.** Summarized molar mass and *Ð* values of the prepared copolymers.

Polymer	*M*_n_ (g/mol)	*M*_w_ (g/mol)	*Ð*
**P1**	9000	19,200	2.1
**P2**	10,800	33,700	3.1
**P3**	15,500	64,300	4.2
**P4**	31,400	138,500	4.4

**Scheme 2.**
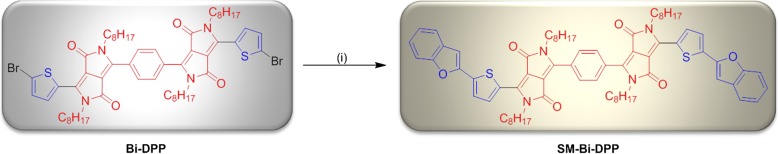
Schematic representation of the synthesis of **SM-Bi-DPP**. Reagents and conditions: (i) Benzofuran-2-ylboronic acid/potassium carbonate/Pd_2_(dba)_3_/((*t*-butyl)_3_PH)BF_4_/tetrahydrofuran/water/25 °C.

Moreover, **Bi-DPP** was investigated as a donor–acceptor small molecule for organic solar cell devices due to several advantages of donor–acceptor small molecules in contrast to donor–acceptor copolymers, like, ease of synthesis and purification (improves fabrication and reproducibility), possessing a better tendency to self-assemble in ordered domains, do not suffer from batch to batch variations or broad molar mass distributions and exhibit no end group contamination.[22] In contrast, it is more challenging to obtain high quality films. According to this, **Bi-DPP** was cross coupled with benzofuran-2-ylboronic acid (**BF**) on each side using a standard Suzuki cross coupling technique to afford **SM-Bi-DPP** as a donor–acceptor small molecule with a yield of 91% with the structure donor-DPP-phenylcore-DPP-donor (Scheme 2). Thereby, the attached **BF** moiety increases the donor strength, maintains a highly conjugated system as well as stabilizes the HOMO of the whole molecule due to the oxygen atom.[22]

### Optoelectronic properties of the prepared Bi-DPP-based conjugated donor–acceptor copolymers and small molecule

UV–vis absorption spectra were recorded for the prepared donor–acceptor copolymers **P1**–**P4** and the donor–acceptor small molecule **SM-Bi-DPP** in chloroform solutions and as thin spin coated films (Figure 2). All prepared polymers without the small molecule exhibit two absorption peaks over the whole spectrum and the specific locations of the absorption maxima are summarized in Table 2. Furthermore, all donor–acceptor-based materials exhibit a broad absorption over the whole visible region past 700 nm in solution, which can be attributed to intramolecular charge transfer between electron accepting and electron donating units (Figure 2(a)).

**Table 2.** Summarized optical and electrochemical properties of the prepared copolymers and the small molecule.

Polymer	*λ*_max_ (nm) solution	*λ*_max_ (nm) film	*λ*_edge_ (nm) film	*E*_g,opt._ (eV)	HOMO_el._ (eV)	LUMO_el._ (eV)	*E*_g,el._ (eV)
**P1**	585, 379	600, 382	693	1.79	−5.34	−3.58	1.76
**P2**	590, 366	600, 372	691	1.79	−5.34	−3.59	1.75
**P3**	594, 368	603, 368	691	1.79	−5.28	−3.55	1.73
**P4**	639, 414	632, 404	742	1.67	−5.07	−3.55	1.52
**SM-Bi-DPP**	605	616	690	1.79	−5.30	−3.49	1.81

The exception represents **P4** with an absorption past 750 nm. The phenomena is due to the fact that the introduced accepting building block **Bi-DPP** interacts with the stronger donor moiety **B** (in contrast to fluorene and silafluorene derivatives) and, consequently, resulting in a lower band gap. Compared to the absorption spectra in solution, the corresponding absorption spectra of the spin coated films exhibit a bathochromic shift of the absorption bands due to the aggregation of the oligomer/polymer chains in the solid state resulting in an increased *π*–*π*-stacking (Figure 2(b)). Furthermore, all absorption features of the prepared polymer films in the visible region were past 800 nm. The absorption onsets (*λ*_edge_) in solution of **P1**, **P2**, **P3**, **P4** and **SM-Bi-DPP** were 693, 691, 691, 742 and 690 nm and the resulting optical band gaps (*E*_g,opt._) were calculated by the equation *E*_g,opt. _= *h* × *c*/*λ*_edge_ to be 1.79, 1.79, 1.79, 1.67 and 1.79 eV, respectively. Moreover, UV−vis emission measurements of the prepared spin coated films were investigated and afford very low emission intensities. The differences in *E*_g,opt._ and as well as absorption maxima indicates that the donor strength is in the following order **B1**, **B2** > **BF** > **S1**, **S2** = **F1**, **F2**. The thicknesses of the spin coated films of **P1**, **P2**, **P3**, **P4** and **SM-Bi-DPP** were determined via AFM to be 40 ± 5, 225 ± 10, 125 ± 25, 425 ± 25 and 185 ± 25 nm, respectively.

Subsequently, the electrochemical properties of the donor–acceptor copolymers and the small molecule of Bi-DPP were investigated by cyclic voltammetry (CV) by utilizing a standard three-electrode setup. Figure 3 shows that all studied samples exhibit quasi reversible redox processes. The onset reduction potentials of **P1**, **P2**, **P3**, **P4** and **SM-Bi-DPP** were −0.73, −0.73, −0.76, −0.76 and −0.82 eV resulting in LUMO energy levels of −3.58, −3.59, −3.55, −3.55 and −3.49 eV by calculation *via* the equation *E*_LUMO _= (−(*E*_onset _− *E*_onset,Fc/Fc+_) − 4.8) eV, respectively. The relatively constant LUMO energy level represents that the LUMO is mainly located on the acceptor moiety **Bi-DPP**. In contrast, the onset oxidation potentials were determined to be 1.03, 1.03, 0.97, 0.76 and 0.99 eV resulting in HOMO energy levels of −5.34, −5.34, −5.28, −5.07 and −5.30 eV by calculation via the equation *E*_HOMO _= (−(*E*_onset _− *E*_onset,Fc/Fc+_) − 4.8) eV, respectively. Consequently, the HOMO is mainly located on the electron donating part of the donor–acceptor copolymer. The calculated electrochemical band gap energies fits nearly with the intended optical band gap energies from the absorption spectra in solution and should be suitable for organic solar cell devices. Normally, a LUMO_donor−acceptor copolymer_−LUMO_PC71BM_ offset of 0.3–0.4 eV is sufficient to ensure efficient charge dissociation in organic solar cell devices.[23] The determined energy levels of the LUMOs are nearly constant, due to utilizing the same electron accepting building block **Bi-DPP**, and proved theoretical by suitable electron transfer to the commercial organic solar cell acceptor material [6,6]-phenyl C_71_ butyric acid methyl ester (PC_71_BM), which is depicted in Figure 4. The HOMO energy levels of the prepared donor–acceptor samples, excluded P4, are in an ideal range to ensure efficient hole transport to PEDOT:PSS, good air stability and open voltage circuits in a final organic solar cell device. In contrast to a donor–acceptor small molecule from Nguyen and co-workers, where a single diketopyrrolopyrrole (DPP) was utilized as the electron accepting part,[22] the novel **SM-Bi-DPP** unit offers a decreased LUMO energy level (from −3.40 to −3.49 eV) as well as a decreased HOMO energy level (from −5.20 to −5.30 eV), which should lead to an increased electron transfer to PC_71_BM as well as an increased hole transfer to PEDOT:PSS in a final organic solar cell device.

The introduction of the novel **Bi-DPP** in donor–acceptor copolymers **P1**–**P4** resulted in a LUMO energy level of −3.55 to −3.59 eV and depended on the utilized donor moiety a HOMO energy level of −5.34 to −5.07 eV. Kazuhito and co-workers [24] and Kanimozhi et al*.* [25] investigated donor–acceptor copolymers with the same donor moieties in combination with the standard single DPP electron accepting unit. Thereby, the single DPP moiety exhibit a LUMO energy level of −3.64 to −3.69 eV, which is slightly lower than those of the presented donor–acceptor copolymers of **Bi-DPP**. When using the fluorene-based donor **F1** and **F2** for donor–acceptor copolymers the HOMO energy level is increased from −5.42 eV (single DPP) to −5.34 eV in the case of the novel **Bi-DPP**. The same trend is visible for the usage of the donor moiety **B1** and **B2** (increasing HOMO energy level from −5.15 eV (single DPP) to −5.07 eV (**Bi-DPP**)).

## Conclusion

In conclusion, four donor–acceptor copolymers as well as one donor–acceptor small molecule were synthesized and studied in regard to the usage of a novel electron accepting building block **Bi-DPP**. The presented **Bi-DPP** was synthesized via a new synthetic pathway by simultaneously building up two DPP moieties step by step starting from a small core unit. The synthesized donor–acceptor copolymers as well as the donor–acceptor small molecule exhibit a broad absorption over the whole visible region, efficient film forming properties, LUMO energy levels from −3.49 to −3.59 eV as well as HOMO energy levels from −5.07 to −5.34 eV resulting in low energy band gaps from 1.52 to 1.81 eV. It could be demonstrated that all prepared donor–acceptor systems represents suitable π-conjugated donor oligomers/polymers for the usage in ‘active materials’ (in combination with PC_71_BM) of organic solar cell devices. Further studies will have to address not only the introduction of different simple electron accepting core moieties, but also the synthesis of a **Bi-DPP** moiety without core unit.

## Funding

This work was financially supported by the Deutsche Forschungsgemeinschaft (DFG) [project number HA6306/3-1].

## Disclosure statement

No potential conflict of interest was reported by the authors.
